# Supplementary material for: How to More Effectively Obtain Ginsenoside Rg5: Understanding Pathways of Conversion
Source: Molecules. 2023 Oct 29;28(21):7313. doi: 10.3390/molecules28217313 (PMC10650205; doi:10.3390/molecules28217313)
Supplement: Supplementary file 1 [file molecules-28-07313-s001.zip › molecules-2615537-supplementary.pdf]

Supplementary Information for

How to more effectively obtain Ginsenoside Rg5: Understanding Pathways of Conversion

Leqin Cheng[1,*], Wei Luo[1], Anqi Ye[1], Yuewei Zhang[1,*], Ling Li[2] and Haijiao Xie[3]

[1] Jilin Institute of Chemical Technology, School of Chemistry and Pharmaceutical Engineering, Jilin 132022, China

[2] Tonghua Bai'aojinsen Biotechnology Co., Ltd, Tonghua, 134000, China

[3] Hangzhou Yanqu Information Technology Co., Ltd., Hangzhou 310003, China

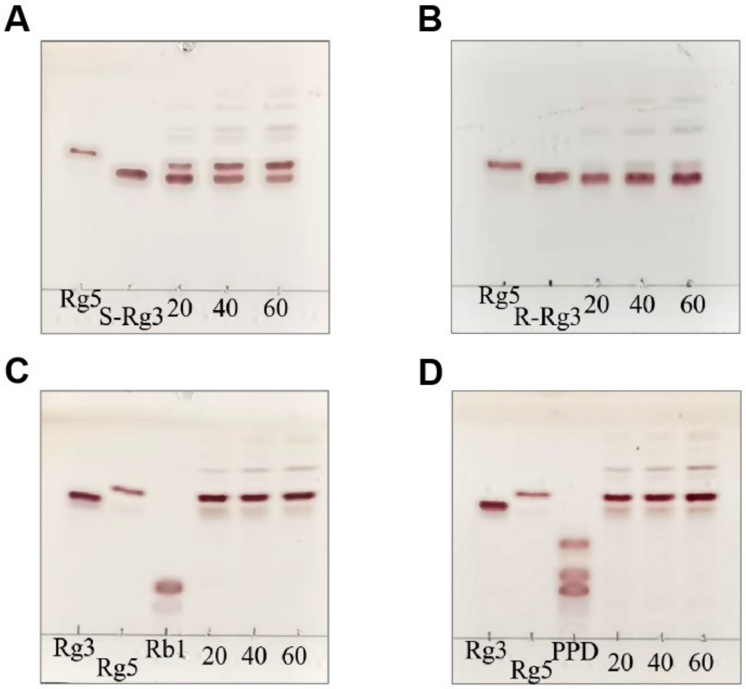

Figure S1. Thin layer chromatography (TLC) analysis of time-course (20 min, 40 min, 60 min) transformation of ginsenoside S-Rg3 (A), R-Rg3 (B), Rb1 (C) and PPD type saponin (D) at 60 ℃ and 300 rpm. Solvent system: $CHCl_3$/MeOH/H_2O (65:35:10, by v/v, lower phase). Spots in the silica gel plates were colored by heating at the 105 ºC after sprayed with 10% sulfuric aqueous solution.

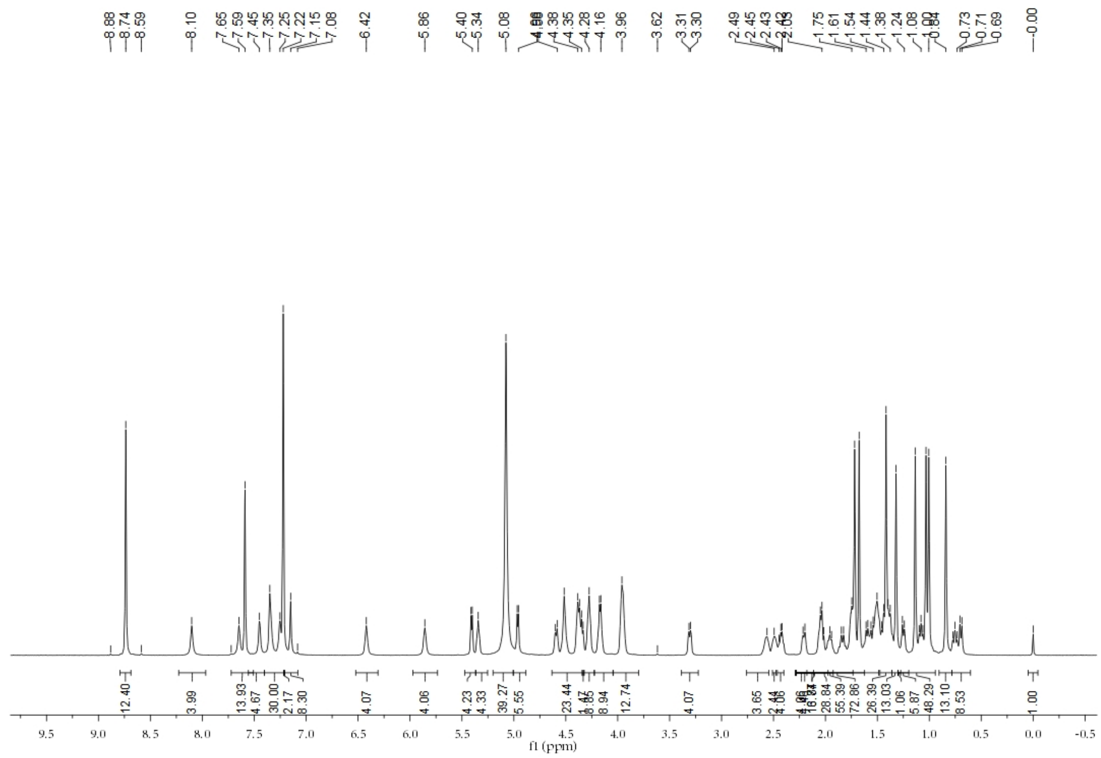

Figure S2. ^{1}H-NMR analysis of R-Rg3 in pyridine-d_5.

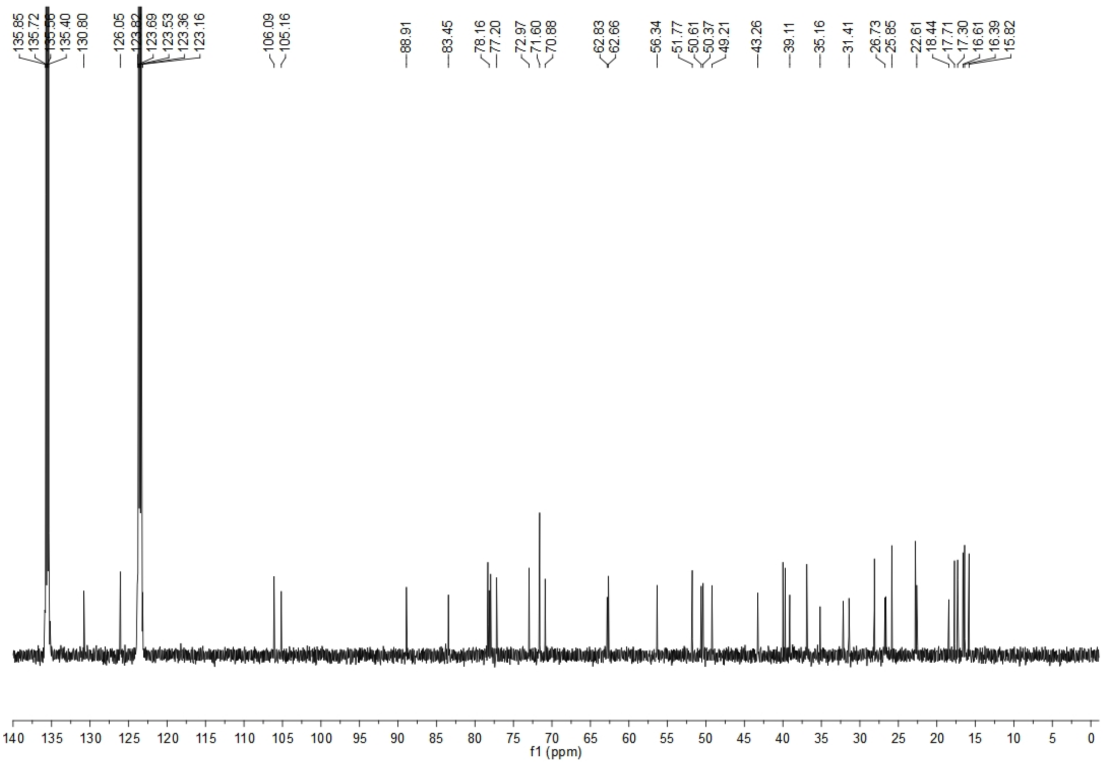

Figure S3. ^{13}C-NMR analysis of R-Rg3 in pyridine-d_5.

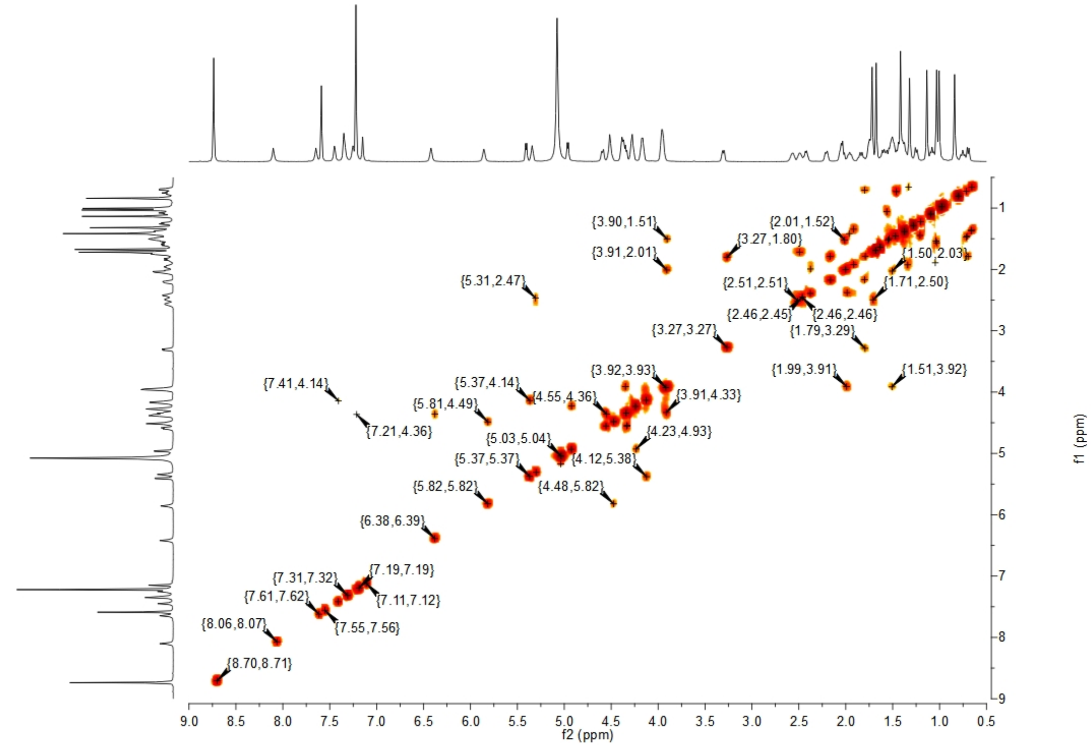

Figure S4. COSY analysis of S-Rg3 in pyridine-d_5.

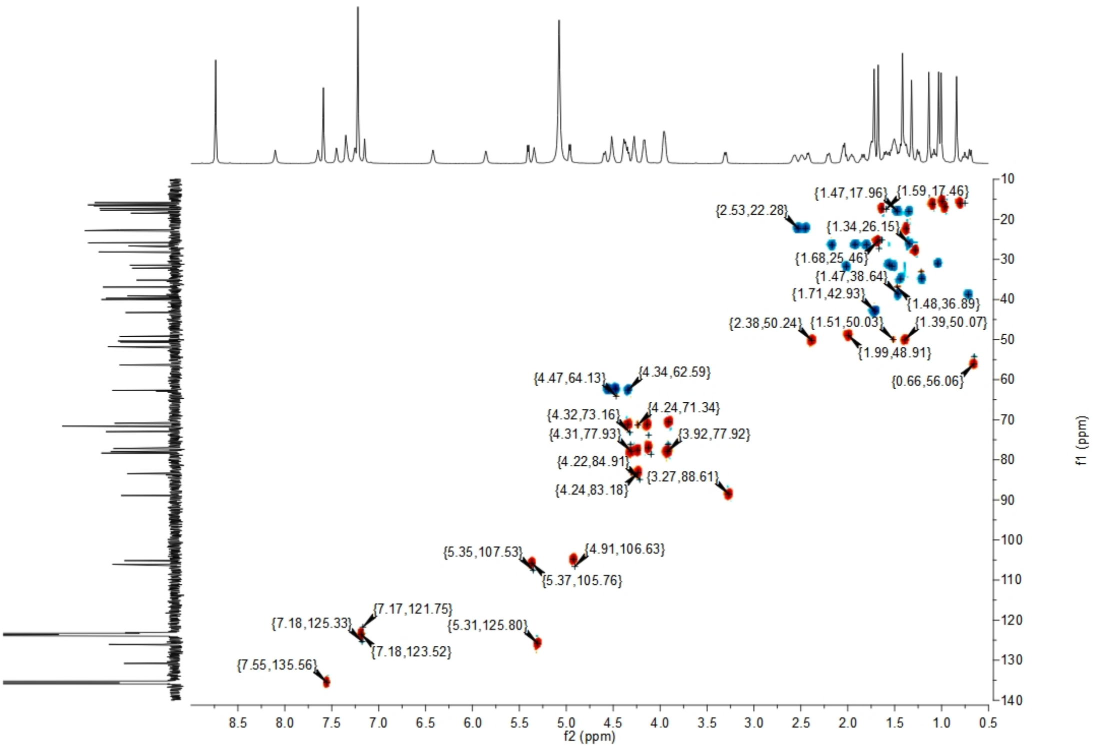

Figure S5. HSQC analysis of R-Rg3 in pyridine-d_5.

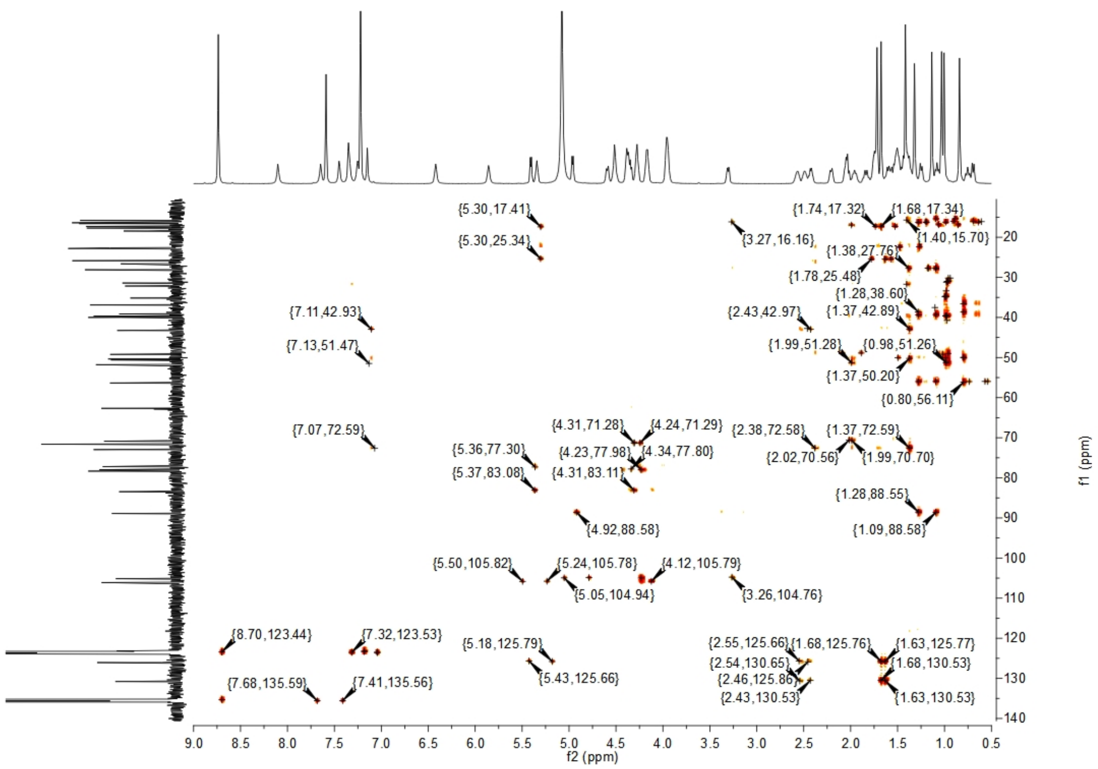

Figure S6. HMBC analysis of R-Rg3 in pyridine-d_5.

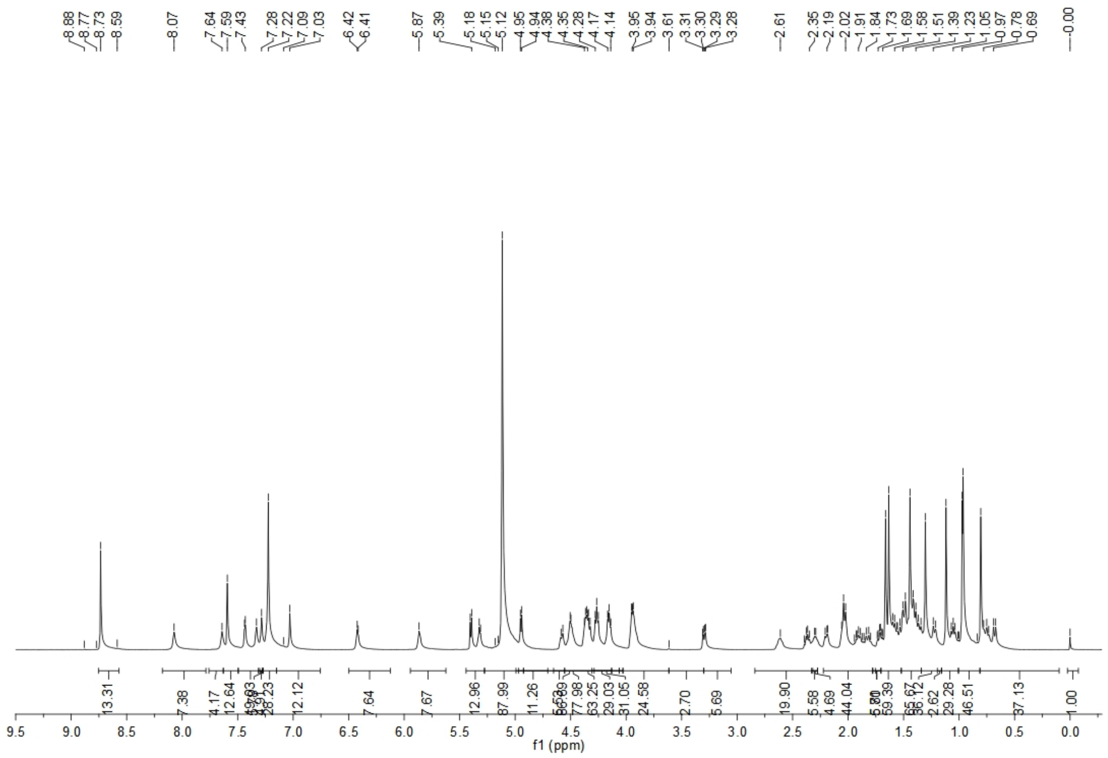

Figure S7. ^{1}H-NMR analysis of S-Rg3 in pyridine-d_5.

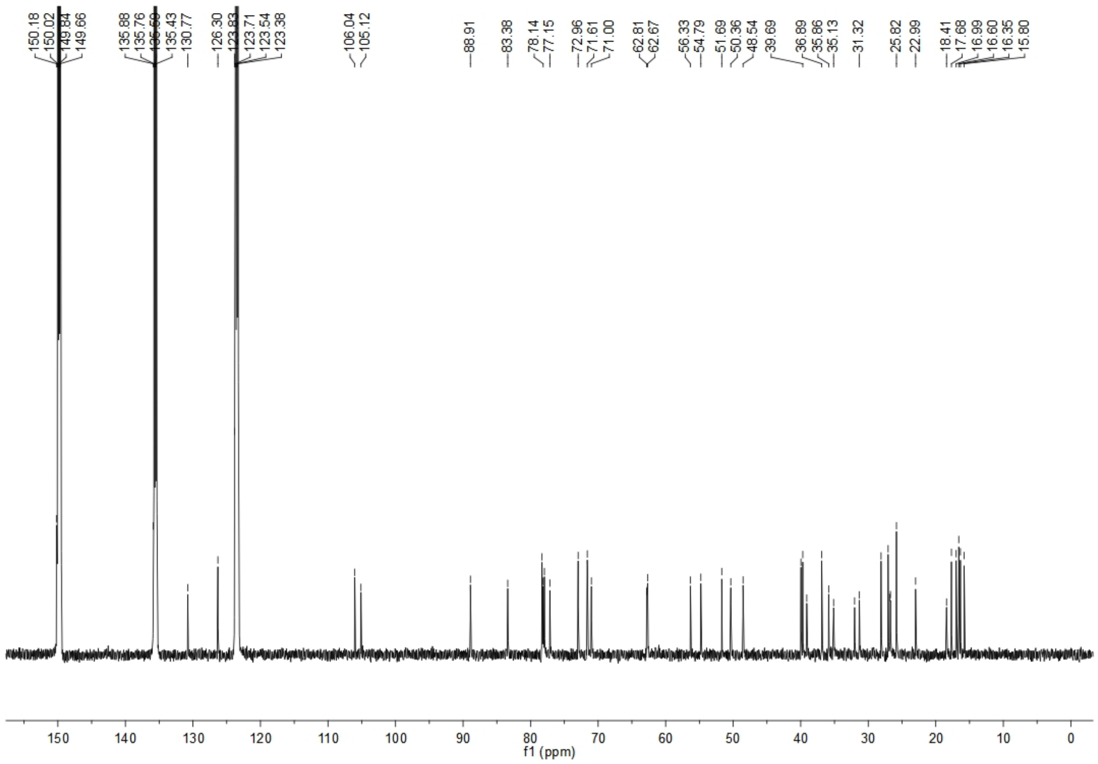

Figure S8. ^{13}C-NMR analysis of S-Rg3 in pyridine-*d*$_5$.

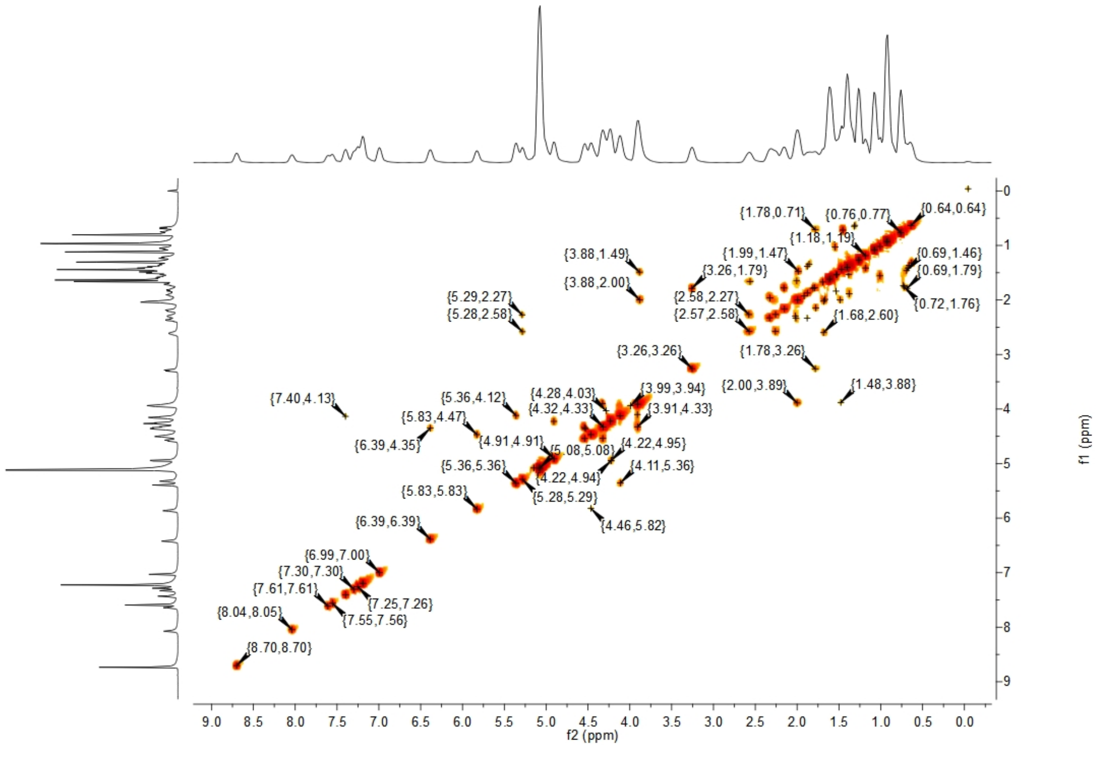

Figure S9. COSY analysis of S-Rg3 in pyridine-*d*$_5$.

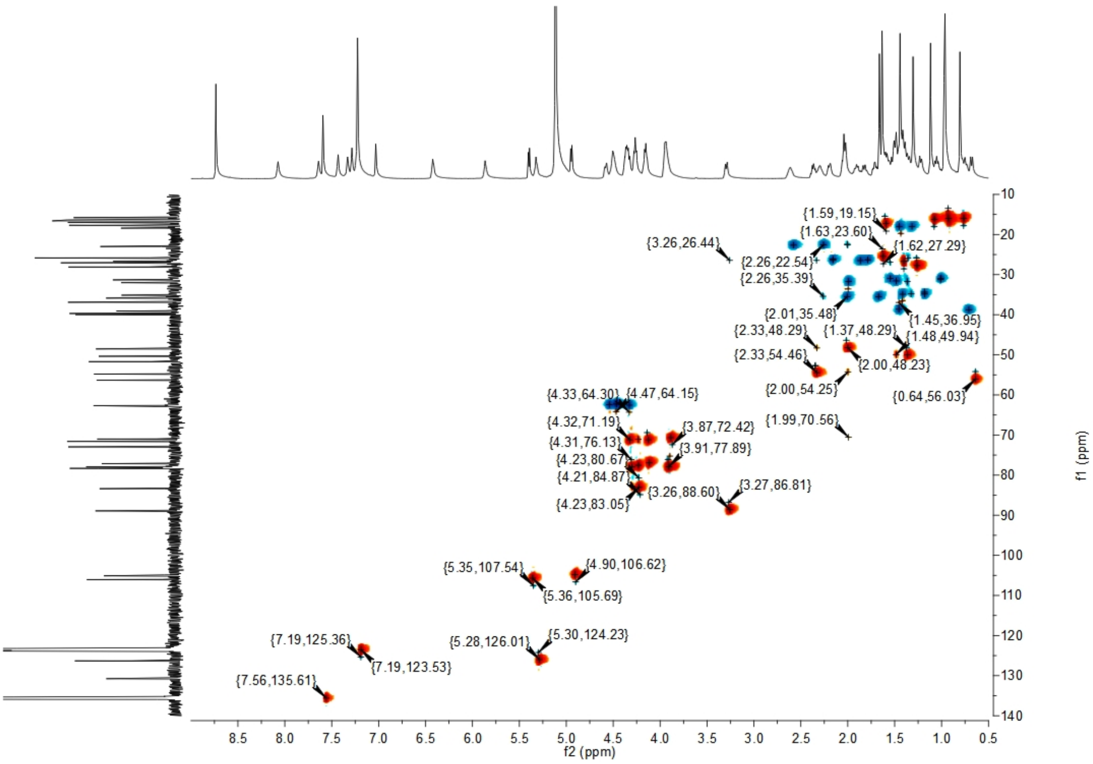

Figure S10. HSQC analysis of S-Rg3 in pyridine-d_5.

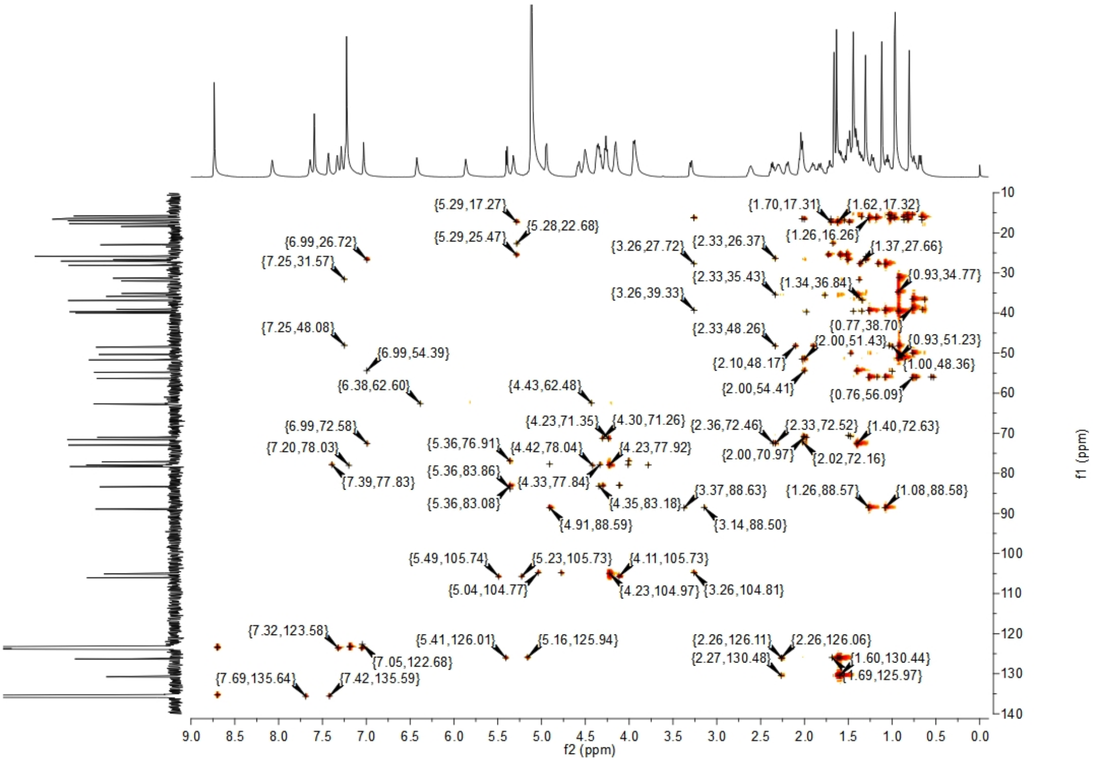

Figure S11. HMBC analysis of S-Rg3 in pyridine-d_5.

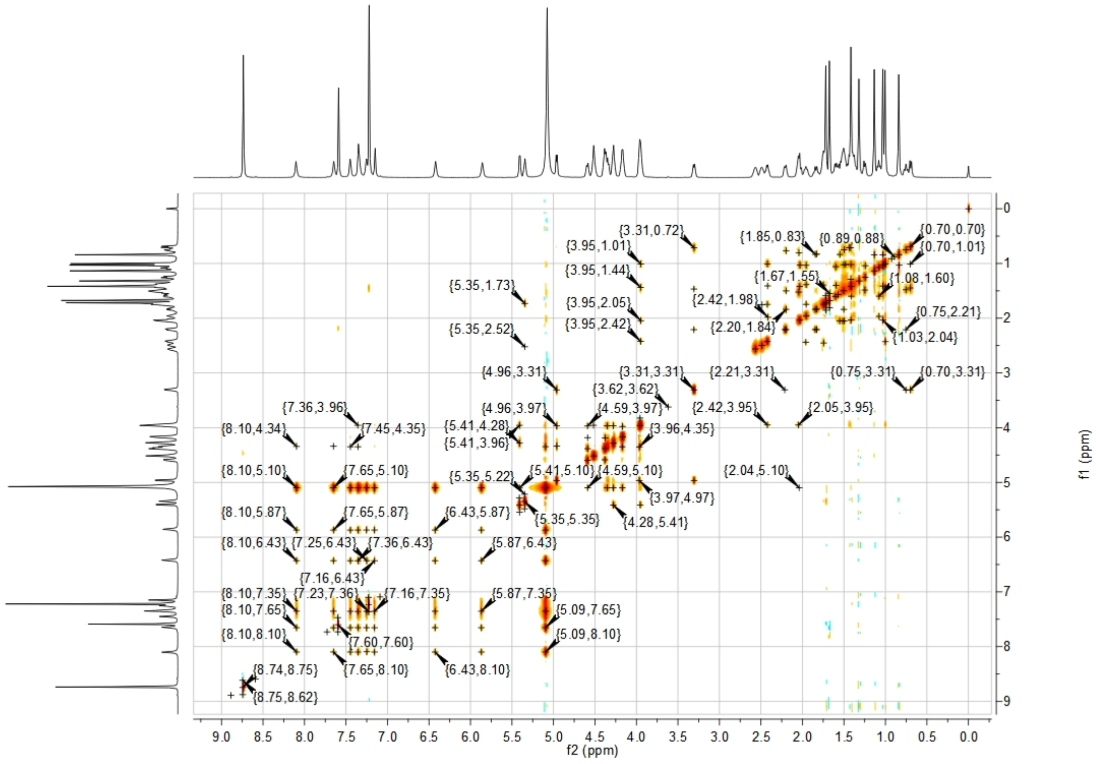

Figure S12. 2D-NOESY analysis of R-Rg3 in pyridine-d_5.

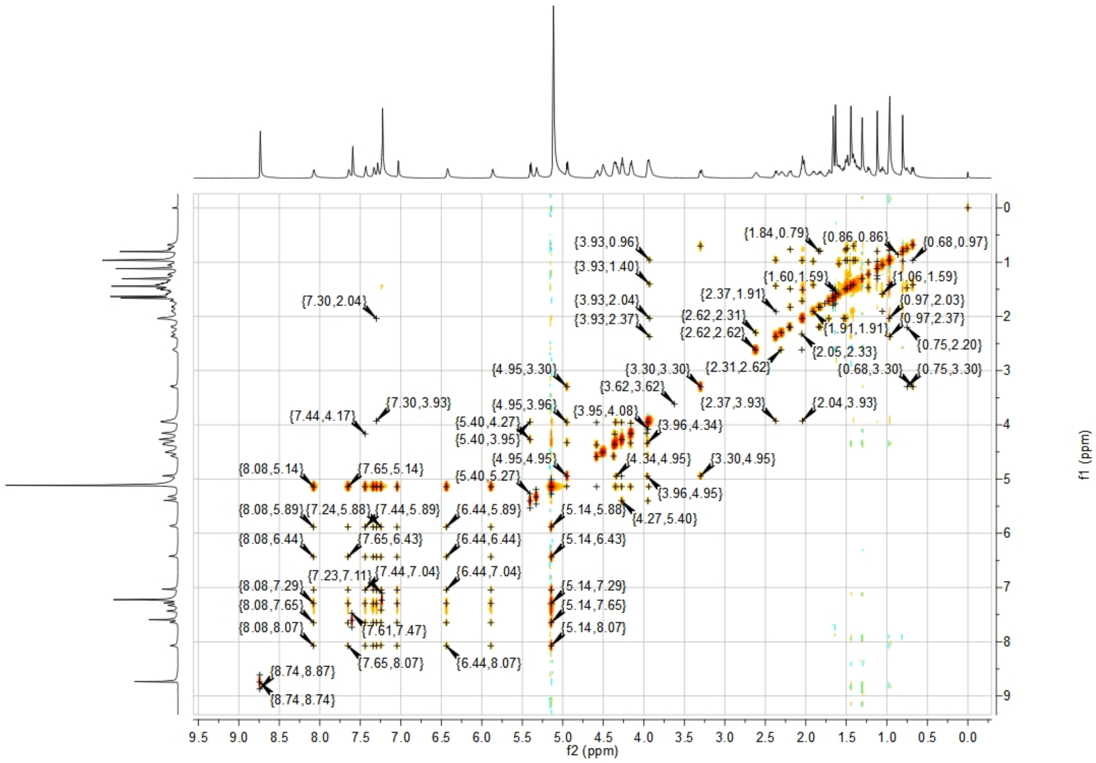

Figure S13. 2D-NOESY analysis of S-Rg3 in pyridine-d_5.

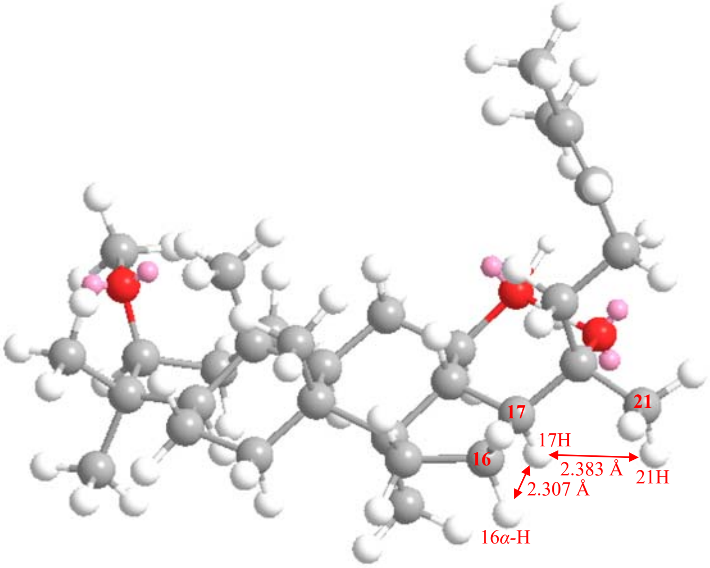

Figure S14. The spatial distance between H17 and H16, H21 in the hydrogen-bonding structure of S-Rg3.

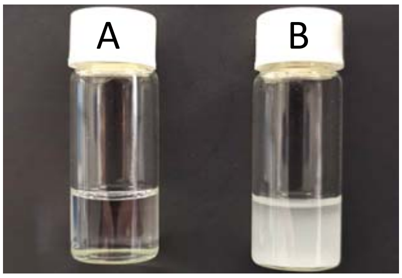

Figure S15. Schematic diagram of S-Rg3 (A) and R-Rg3 (B) solution (1 mg/mL methanol solution).

Table S1. Conversion rate and yield of ginsenoside Rg5 prepared from different raw materials.

Raw materials	Time (min)	Conversion rate (%)	Yield of S-Rg3 (%)	Yield of R-Rg3 (%)	Yield of Rg5 (%)
S-Rg3	20	38.97	-	-	18.81
R-Rg3	20	10.59	-	-	3.04
Rb1	20	100	6.80	0.83	48.92
PPD	20	100（Rb1）<br>100（Rc）<br>100（Rb2）<br>100（Rb3）<br>100（Rd）	6.78	0.32	45.42
PPD (amplification experiment)	20	100（Rb1）<br>100（Rc）<br>100（Rb2）<br>100（Rb3）<br>100（Rd）	4.38	0.38	47.71

Table S2. Free energetics (in kcal/mol) for the three conformation for Rb1, R-Rg3 and S-Rg3 at M06-2X/def2-SV(P)/SMD (methanol) level.

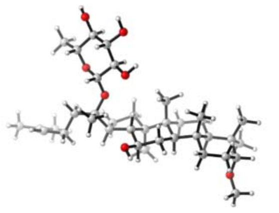	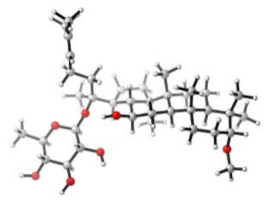	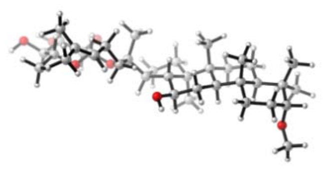
Rb1_Conformation 1 (8.23)	Rb1_Conformation 2 (0.00)	Rb1_Conformation 3 (12.47)
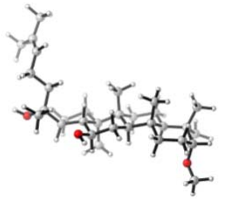	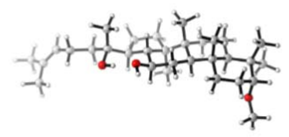	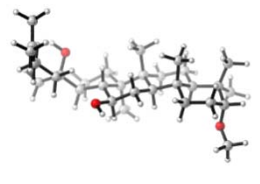
R-Rg3_Conformation 1 (8.34)	R-Rg3_Conformation 2 (0.00)	R-Rg3_Conformation 3 (7.38)
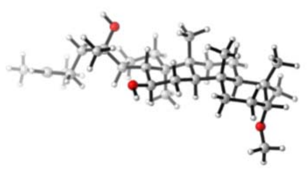	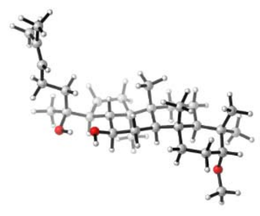	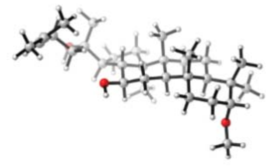
S-Rg3_Conformation 1 (7.35)	S-Rg3_Conformation2 (0.00)	S-Rg3_Conformation 3 (5.90)

Table S3. Specific parameters for ^{1}H-NMR (Proton Nuclear Magnetic Resonance)

No.	parameters	R-Rg3	S-Rg3
1	Spectrometer frequency (MHz)	600.13	600.13
2	Solvent used	pyridine-d_5	pyridine-d_5
3	Sample concentration (mg/mL)	40	40
4	Temperature (°C)	22	22
5	Number of scans	8	8
6	Pulse width (π/2 pulse)	12.06	12.06
7	Relaxation delay (D1)	1.0	1.0
8	Acquisition time	3.1457	3.1457
9	Data processing and phase correction details	The spectra were Fourier transformed, automatic phased, and manually baseline corrected. TMS was selected for peak position calibration (reference calibration) and set its chemical shift to zero. The integral of the peak that can determine the number of hydrogen atoms was selected for reference integral, and then output the spectrum.	

Table S4. Specific parameters for ^{13}C-NMR (Carbon-13 Nuclear Magnetic Resonance)

No.	parameters	R-Rg3	S-Rg3
1	Spectrometer frequency (MHz)	150.9	150.9
2	Solvent used	pyridine-d_5	pyridine-d_5
3	Sample concentration (mg/mL)	40	40
4	Temperature (°C)	22	22
5	Number of scans	439	512
6	Pulse width (π/2 pulse)	12.43	12.43
7	Relaxation delay (D1)	2.0	2.0
8	Acquisition time	0.8061	0.8061
9	Data processing and phase correction details	The spectra were Fourier transformed, automatic phased, and manually baseline corrected. The peak position (reference correction) select the pyridine-d_5 for correction, and then output the spectrum.	

Table S5. Specific parameters for ^{1}H-^{1}H COSY (Correlation Spectroscopy)

No.	parameters	R-Rg3	S-Rg3
1	Spectrometer frequency (MHz)	600.13, 600.13	600.13, 600.13
2	Solvent used	pyridine-d_5	pyridine-d_5
3	Sample concentration (mg/mL)	40	40
4	Temperature (°C)	22	22
5	Number of scans	8	8
6	Pulse width (π/2 pulse)	12.06	12.06
7	Relaxation delay (D1)	1.5	1.5
8	Acquisition time	0.0625	0.0325
9	Data processing and phase	The spectra were Fourier transformed, manually phased, and	

	correction details	baseline corrected. TMS was selected for peak position calibration (reference calibration) and set its chemical shift to zero (f1) and zero (f2), and then output the spectrum.

Table S6. Specific parameters for ^{1}H-^{1}H NOESY (Nuclear Overhauser Effect Spectroscopy)

No.	parameters	R-Rg3	S-Rg3
1	Spectrometer frequency (MHz)	600.13, 600.13	600.13, 600.13
2	Solvent used	pyridine-d_5	pyridine-d_5
3	Sample concentration (mg/mL)	40	40
4	Temperature (°C)	22	22
5	Number of scans	4	4
6	Pulse width (π/2 pulse)	12.06	12.06
7	Relaxation delay (D1)	1.5	1.5
8	Acquisition time	0.1311	0.1311
9	Spectral width	7812.5, 8403.4	8196.7, 8403.4
10	mixing time for NOESY (ms)	800	800
11	Data processing and phase correction details	The spectra were Fourier transformed, manually phased, and baseline corrected. TMS was selected for peak position calibration (reference calibration) and set its chemical shift to zero (f1) and zero (f2), and then output the spectrum.	

Table S7. Specific parameters for ^{1}H-^{13}C HSQC (Heteronuclear Single Quantum Coherence)

No.	parameters	R-Rg3	S-Rg3
1	Spectrometer frequency for both ^{1}H and ^{13}C nuclei (MHz)	150.9, 600.13	150.9, 600.13
2	Solvent used	pyridine-d_5	pyridine-d_5
3	Sample concentration (mg/mL)	40	40
4	Temperature (°C)	22	22
5	Number of scans	8	8
6	Pulse widths for both ^{1}H and ^{13}C	12.06	12.06
7	Relaxation delay (D1)	1.5	1.5
8	Acquisition time	0.0655	0.0655
9	Data processing and phase correction details	The spectra were Fourier transformed, manually phased, and baseline corrected. The peak position (reference correction) select the pyridine-d_5 for correction, and then output the spectrum.	

Table S8. Specific parameters for ^{1}H-^{13}C HMBC (Heteronuclear Multiple Bond Correlation)

No.	parameters	R-Rg3	S-Rg3
1	Spectrometer frequency for both ^{1}H and ^{13}C nuclei (MHz)	150.9, 600.13	150.9, 600.13
2	Solvent used	pyridine-d_5	pyridine-d_5
3	Sample concentration (mg/mL)	40	40

4	Temperature (°C)	22	22
5	Number of scans	8	8
6	Pulse widths for both ^{1}H and ^{13}C	12.06	12.06
7	Relaxation delay (D1)	1.5	1.5
8	Acquisition time	0.1249	0.1249
9	long-range coupling delay (*J*-coupling delay)(Hz)	8	8
10	Data processing and phase correction details	The spectra were Fourier transformed, manually phased, and baseline corrected. The peak position (reference correction) selects 27H and 24C correlation peak for correction, and then outputs the spectrum.	
